# Cdk5 Inhibitory Peptide Prevents Loss of Dopaminergic Neurons and Alleviates Behavioral Changes in an MPTP Induced Parkinson’s Disease Mouse Model

**DOI:** 10.3389/fnagi.2018.00162

**Published:** 2018-06-01

**Authors:** Rongni He, Wei Huang, Yaowei Huang, Miaojing Xu, Pingping Song, Yinwei Huang, Huifang Xie, Yafang Hu

**Affiliations:** ^1^Department of Neurology, Nanfang Hospital, Southern Medical University, Guangzhou, China; ^2^Department of Neurology, Zhujiang Hospital, Southern Medical University, Guangzhou, China

**Keywords:** AAV9, cyclin-dependent kinase 5, CIP, Parkinson’s disease, MPTP

## Abstract

Parkinson’s disease (PD) is one of the most affected neurodegenerative diseases in the world. Deregulation of cyclin-dependent kinase 5 (Cdk5) is believed to play an important role in neurodegenerative diseases including PD. p25 is a cleavage peptide of p35, a physiologic activator of Cdk5. p25 combines to Cdk5 and leads to the hyperactivity of Cdk5, which in turn hyperphosphorylates downstream substrates and leads to neuroinflammation and apoptosis of neurons. Previously, we have demonstrated that adeno-associated virus serotype-9 (AAV9) mediated Cdk5 inhibitory peptide (CIP) inhibits the activity of Cdk5/p25 complex and alleviates pathologic and behavioral changes in Alzheimer’s disease mouse model. In this study, we evaluated whether AAV9-CIP protected dopaminergic (DA) neurons in 1-methyl-4-phe-nyl-1,2,3,6-tetrahydropyridine-probenecid (MPTP/p) induced PD mouse model. The data showed that administration of AAV9-CIP by intracerebroventricular injection 1 week before MPTP/p exposure protected loss of DA neurons in substantia nigra compact of the model mice. Importantly, AAV9-CIP also alleviated the motor and anxiety-like symptoms of the disease animals. In summary, AAV9 mediated CIP might be a potential intervention for PD.

## Introduction

Parkinson’s disease (PD) is the second most popular neurodegenerative disease, which manifests as rigid, bradykinesia, myotonia, resting tremor, and ataxia (Brucker and Kalra, 2017; Ellis and Fell, 2017). PD is characterized by progressive apoptosis of dopaminergic (DA) neurons in substantia nigra compact (SNpc; Ben-Shlomo, 2008). Though the clinical symptoms can be controlled by levodopa, dopamine decarboxylase inhibitor, DA agonist, and deep brain stimulation, neuronal degeneration continues to progress (Lang and Lozano, 1998).

Cyclin-dependent kinase 5 (Cdk5), a proline-directed serine/threonine protein kinase, is essential for regulating mammalian central nervous system (CNS) development, which is dependent on binding and activating by p35 or p39 (Kanungo et al., 2009; Shupp et al., 2017). Deregulation of Cdk5 is reported to be involved in many neurodegenerative diseases, such as Alzheimer’s disease (AD) and PD. Under stress conditions such as oxidative stress and amyloid presence, p35 is cleaved into p25. Compared to p35, p25 highly binds with Cdk5. Cdk5/p25 complex more widely distributes in cytosol and hyperphosphorylates more substrates related to the pathogenesis of neurodegenerative disease including PD (Patrick et al., 1999; Sahlgren et al., 2006). Studies have showed that Cdk5/p25 complex plays a pivotal role in the DA neurons apoptosis in SNpc, which is responsible for the symptoms of PD patients and animal models (Avraham et al., 2007).

Several truncated peptides of p35, such as Cdk5 inhibitory peptide (CIP, amino acid, aa, 154–279 of p35, 125 aa; Zheng et al., 2005), p5 (aa 245–277 of p35, 24 aa; Zheng et al., 2010), p10 (aa1–98 of p35), and p10’ (aa1–135 of p35; Zhang et al., 2012; Amin et al., 2016) are reported to specifically suppress the activity of Cdk5/p25 complex. In contrast to other Cdk5 inhibitors, roscovitine or Cdk5 siRNA (Guan et al., 2010; Gutiérrezvargas et al., 2015; Posadaduque et al., 2017), such peptides have limited effect on physiological activity of Cdk5/p35. We and others have demonstrated that TFP5, which is modified at the C-terminus of P5 by Tet peptide to facilitate across the blood–brain barrier and labeled with fluorescein isothiocyanate at the N-terminus of P5, provides neuroprotection in AD mouse model (Shukla et al., 2013) and 1-methyl-4-phenyl-1,2,3,6-tetrahydropyridine (MPTP) induced PD mouse models (Binukumar et al., 2015; Zhang et al., 2016b). Transgenic overexpression of CIP reduces p25 mediated neurodegeneration in the CIP–p25 tetra transgenic (TetraTg) mice (Sundaram et al., 2013). To overcome the limitation of CIP to cross the brain–blood barrier, we used CNS-directed AAV serotype 9 as the delivery system of CIP to assure stable gene expression in CNS (Gonçalves, 2005; Meredith et al., 2008; Weinberg et al., 2013; Dashkoff et al., 2016; Merkel et al., 2016). Adeno-associated virus serotype-9 (AAV9)-GFP-CIP alleviates pathologic and behavioral changes in AD mouse model (He et al., 2017). Here, we would like to detect whether AAV9-GFP-CIP had neuroprotection in an MPTP-probenecid (MPTP/p) induced PD mouse model.

In the present study, MPTP/p induced PD mouse model was used (Meredith et al., 2008). AAV9-GFP-CIP was administrated via intracerebroventricular (ICV) injection. Compared to control vector, AAV9-GFP-CIP reduced loss of DA neurons and alleviated the motor and non-motor symptoms of the MPTP/p induced PD model mice. Our data suggest that inhibition of Cdk5 dysfunction by CIP may become a potential therapy for PD.

## Materials and Methods

### Animals

Eight-week-old male C57BL/6 mice were purchased from the Laboratory Animal Center of Southern Medical University. Animals were housed in standard cages under 12 h light/dark cycle, standard conditions of temperature (∼22°C), and humidity (30–50%). Mice were accessed to food and water *ad libitum*. All animal experiments were performed according to approved protocols by the University Committee on Animal Care of Southern Medical University, China, and conducted in accordance with the Guide for the Care and Use of Laboratory Animals.

### Establishment of an MPTP/p Induced PD Mouse Model

**FIGURE 1:**
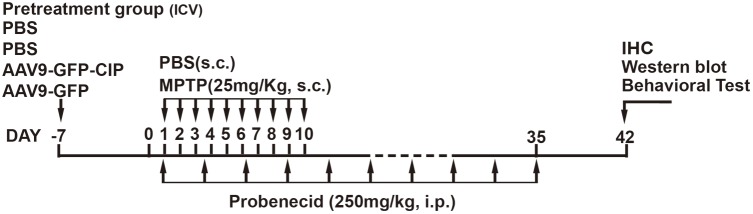
Schematic chart of drug treatments in mice. Forty-eight mice were randomized into four treatment groups with 12 mice in each group, including (1) control group, PBS; (2) PBS group, PBS pretreatment + MPTP/p insult; (3) GFP group, AAV9-GFP pretreatment + MPTP/p insult; and (4) CIP group, AAV9-GFP-CIP pretreatment + MPTP/p insult. The animals were pre-treated with PBS, AAV9-GFP, and AAV9-GFP-CIP 7 days before initiation of MPTP/p insult. Then behavioral test, western blot, and IHC analysis were performed on day 42. MPTP/p, 1-methyl-4-phenyl-1,2,3,6-tetrahydropyridine/phenobencid.

The MPTP/p induced PD model was modified based on reference described (Meredith et al., 2008). In brief, 9-week-old mice were treated with MPTP hydrochloride (MPTP-HCL, Sigma, St. Louis, MO, United States) and probenecid (Sigma, St. Louis, MO, United States). Mice were subcutaneously (s.c.) injected with 100 μl MPTP (freshly solved in PBS as 25 mg/kg) daily for 10 consecutive days; 100 μl of probenecid resolved in Tris-HCL, pH7.4 as 250 mg/kg were injected intraperitoneally (i.p.) twice per week for 5 weeks. Control mice were injected with PBS and probenecid in the same volume. The schematic chart was indicated as in **Figure 1**.

### AAV9 Vectors’ Pretreatment via ICV Injection

Vector AAV9-GFP-CIP, expressing N-terminal fused CIP driven by the human SYN-1 promoter, and the control vector AAV9-GFP were prepared (Healthgen Biotechnology Corp., Wuhan, China) and diluted in PBS at a final concentration of 10^12^ vector genome (vg)/ml as previously reported (He et al., 2017).

Forty-eight 8-week-old male mice were randomly divided into four groups with 12 mice in each group and receiving 2 μl virus treatment via ICV injection 1 week early before MPTP/p treatment as displayed in **Figure 1**: (1) control group, PBS; (2) PBS group, PBS+MPTP/p insult; (3) GFP group, AAV9-GFP pretreatment + MPTP/p insult; and (4) CIP group, AAV9-GFP-CIP pretreatment + MPTP/p insult. The stereotaxic coordinates for ICV injection were 1.0 mm lateral, 0.5 mm posterior to the bregma and 2.5 mm deep from the dural surface (Paxinos and Franklin, 2001). **Figure 1** shows the schematic chart of pretreatment paradigm.

### Western Blot

Western blot analysis of the lysates from mice brain was routinely performed as described (Sundaram et al., 2012). Nigral tissue samples were extracted by using a 1-mm-diameter needle which dissected the SNc area as described (Smith et al., 2003). In brief, 7 days after last probenecid injection, mice were deeply anesthetized by 2% pentobarbital sodium (60 mg/kg, RWD, Shenzhen, China). A 2-mm coronal slab of the midbrain was immediately isolated and frozen at -80°C. Nigral tissues were homogenized in Tris-NaCl lysate buffer consisting 5 mM EDTA, 0.1% Nonidet P-40, 5 mM DTT, 10 mM NaF, 1 μg/ml aprotinin, 1 μg/ml leupeptin, 1 mM PMSF, and 50 mM Tris-HCl at pH 7.5. Anti-tyrosine hydroxylase (TH; Millipore, Billerica, MA, United States), anti-p-MEF2D (Thermo Fisher, Waltham, MA, United States), anti-p35 (Santa cruz, CA, United States), and anti-β-actin (Proteintech, China) primary antibodies were diluted at 1:1000. Horseradish-peroxidase-labeled secondary antibodies (Boster, China) were used at 1:1000 dilutions. Signals were detected by Kodak *in vivo* imaging system FX Pro suite (Kodak, Rochester, NY, United States).

### Histochemical and Immunofluorescence Assessment

Immunohistochemistry and immunofluorescence were performed as reported (Sundaram et al., 2012; Chen et al., 2017). In brief, mice were deeply anesthetized by 2% pentobarbital sodium and transcardially perfused with 4°C PBS and 4% paraformaldehyde. Brains were extracted and fixed in 4% paraformaldehyde for 24 h; 4 μm sections of the midbrain and the striatum were sliced by Leica RM vibratome (Leica Microsystems, Heidelberg, Germany). Slices for immunohistochemistry were incubated with anti-TH antibody (Millipore, Billerica, MA, United States). Goat against rabbit secondary antibody was from ZSGB-BIO (Beijing, China). Slices for immunofluorescence were incubated with anti-TH (Millipore, Billerica, MA, United States) or anti-GFP antibody (Aves Labs, Washington, DC, United States). Secondary antibodies were goat against rabbit antibody (Alexa Fluor^®^ 594) and goat against chicken antibody (Alexa Fluor^®^ 488). The nucleus was stained by DAPI (Boster, China). Images were obtained by research inverted system microscope (Olympus, Tokyo, Japan) or laser scanning confocal microscope (Olympus, Tokyo, Japan). The gray level was analyzed by ImageJ (National Institutes of Health, Bethesda, MD, United States) and the number of TH positive neurons was counted blindly by independent investigators.

### Behavioral Studies

Fifteen-week-old mice were housed in test room and habituated for 24 h. All tests were performed consecutively as described previously (He et al., 2017).

#### Rotarod Test

Rotarod test was performed using an accelerating rotarod (TSE, Homburg, Germany). After acclimatization training, mice were placed on a rotating drum for three trials. The speed of the rotarod accelerated from 4 to 40 rpm within 5 min. The duration of each animal maintained on the rod was recorded.

#### Open Field Test

A 40 cm × 40 cm × 40 cm activity chamber was used to test exploratory behavior and general activity of the mice. Mice were placed inside the chamber and monitored for 30 min with an overhead camera connected to software (TSE).

#### Elevated Plus Maze

Elevated plus maze instrument (TSE) was made of acrylic material with two open arms (30 cm × 5 cm), two closed arms (30 cm × 5 cm × 20 cm), and an open square (5 cm × 5 cm) in the center. Maze was 50 cm above the floor. Mice were put in the central area with the head toward the same closed arm. The times of each mouse entering the open arms and the duration of the mouse reminded in open arms were recorded for 5 min.

### Statistics

All values are presented as means ± SD. Dates of behavioral tests are subjected to one-way ANOVA followed by least significant difference test by using SPSS 16.0 statistical program (SPSS, Chicago, IL, United States). *P*-value < 0.05 was considered to indicate statistical significance.

## Results

### Distribution of Expressed Protein by ICV Injection of AAV9 Virus

**FIGURE 2:**
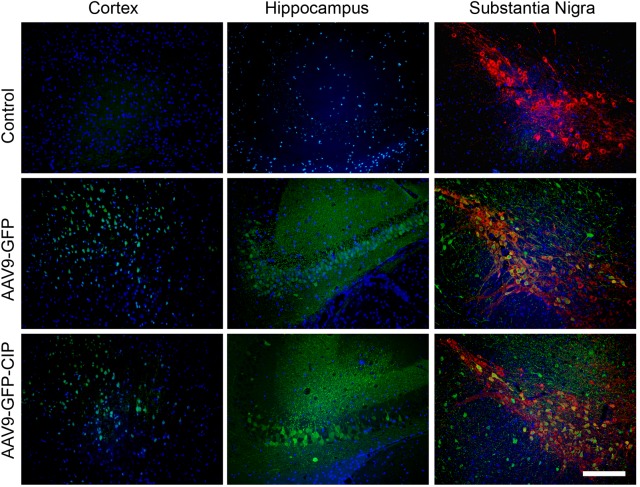
Distribution of AAV9 vectors in brain after intracerebroventricular injection. Immunofluorescence images of cortex, hippocampus, and midbrain from mice 1 week after administration of PBS, AAV9-GFP, or AAV9-GFP-CIP. GFP was immunostained with anti-GFP and goat anti-chicken secondary antibody (Alexa Fluor^®^ 488, Green). TH was immunostained with anti-TH and goat anti-rabbit secondary antibody (Alexa Fluor^®^ 594, red). Nuclei were counterstained with DAPI (blue). Green fluorescence were detected in GFP group and CIP group when there is no green fluorescence in control group.

To check whether ICV injected AAV9 virus is able to express target protein in predicted brain area, the expression of GFP in the brain of mice was analyzed 1 week after ICV injection of AAV9-GFP and AAV9-GFP-CIP. As shown in **Figure 2**, GFP was detected in the cortex, hippocampus, and midbrain sections in virus injected groups, but not in the PBS control group. Co-expression of GFP and TH was observed in the substantia nigra cells. This data confirmed that AAV9 via ICV injection expresses target protein in wide area including the midbrain.

### AAV9-GFP-CIP Reduces the Phosphorylation of MEF2D and DA Neurons’ Death in an MPTP/p Induced PD Mouse Model

**FIGURE 3:**
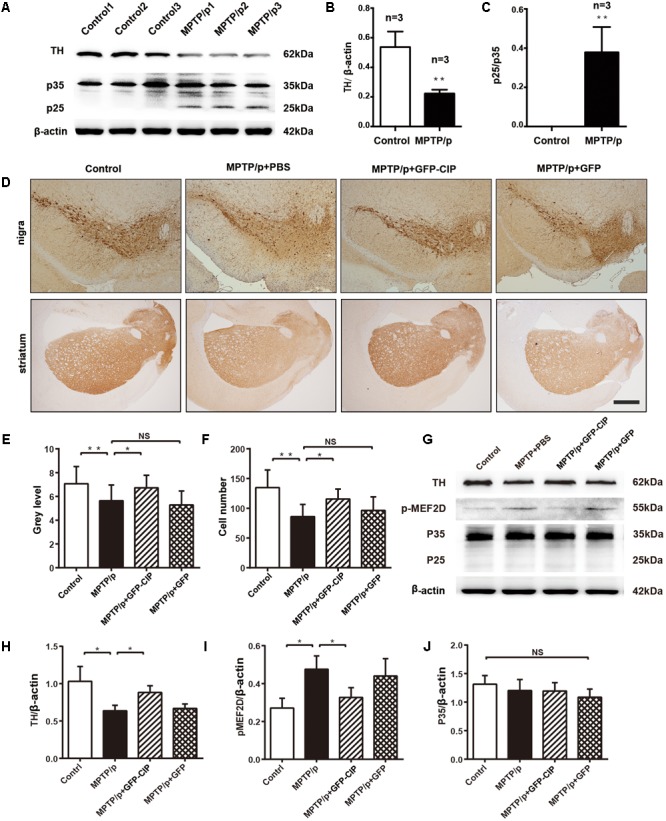
AAV9-GFP-CIP reduced DA neuronal loss and the level of phosphorylated MEF2D in MPTP/p mouse model. Mice were administrated with PBS, AAV9-GFP-CIP, or AAV9-GFP via intracerebroventricular injection 1 week before MPTP/p insult. The MPTP/p induced PD model was established by subcutaneously injection of 100 μl of MPTP (25 mg/kg) daily for 10 days and intraperitoneally injection of 100 μl of probenecid (250 mg/kg) twice per week for 5 weeks. **(A)** Western blot analysis of brain lysates from mice administrated with MPTP/p or PBS control mice were performed using anti-β-actin, anti-p35 (detection of C-terminus of p35), and anti-tyrosine hydroxylase (TH) primary antibodies. Protein sizes were indicated along the right side. **(B)** Relative ratio of TH intensity per β-actin intensity. The relative level of tyrosine hydroxylase was significantly decreased in MPTP/p induced mice compared to control mice (*p* < 0.05). **(C)** Relative ratio of p25 intensity per p35 intensity. A high level of p25 was detected in MPTP/p mouse model, while there was no p25 detected in control mice (*n* = 3). **(D)** Immunohistochemistry images of midbrain striatum and substantia nigra from control group, PBS group, GFP group, and CIP group mice. The sections were incubated with anti-TH primary antibody and goat anti-rabbit secondary antibody. **(E)** The mean density of TH in striatum from immunohistochemistry. The mean density of TH in the PBS group (5.638 ± 0.33 *n* = 6) was decreased when compared to the control group (7.074 ± 0.40 *n* = 6, ^∗∗^*p* < 0.01). The alteration was alleviated in GFP-CIP treatment group (6.725 ± 0.30 *n* = 6), compared to PBS treated group (^∗^*p* < 0.05), while there was no difference between PBS treated group and GFP treated group (5.286 ± 0.34 *n* = 6), labeled NS. **(F)** The number of TH positive neurons in nigra from immunohistochemistry. Neurons were significantly decreased in MPTP/p+PBS group (86.17 ± 8.31 *n* = 6), compared to control group (134.7 ± 12.06 *n* = 6, ^∗∗^*p* < 0.01). The TH positive neurons were significantly restored in CIP treated group (115.5 ± 6.93 *n* = 6, ^∗^*p* < 0.05). No difference was observed between the GFP (96.33 ± 9.36 *n* = 6) group and PBS group (86.17 ± 8.31), labeled NS. **(G–J)** Western blot analysis of phosphorylated MEF2D. p-MEF2D was immunostained with anti-p-MEF2D and goat anti-rabbit secondary antibody. The phosphorylation of MEF2D was significantly increased in the PBS and GFP group, and decreased in the CIP group when compared to the PBS group (^∗^*p* < 0.05 *n* = 3).

We and others have demonstrated that inhibition of Cdk5 hyperactivity by TFP5 provides DA neuroprotection in acute and subacute MPTP induced mouse models (Binukumar et al., 2015; Zhang et al., 2016b). Here, we want to evaluate the effect of Cdk5 inhibition by CIP in an MPTP/p induced PD mouse model, in which daily MPTP injection was for 10 days and probenecid was injected over 5 weeks (twice per week) to block the rapid clearance of MPTP toxin and its metabolites (Gonçalves, 2005). As indicated in **Figures 3A,B**, western blot analysis with anti-TH antibody clearly indicated that TH level significantly decreased at one 1 checkpoint after the last injection of probenecid. MPTP/p exposure led to loss of TH containing DA neurons in nigra and striatum, as indicated in **Figure 3D** (MPTP/p + PBS line). These data indicated that a PD mouse model had established successfully. In addition, the level of p25 in MPTP/p mouse model was significantly higher than the control mouse (*p* < 0.01, *n* = 3; **Figures 3A,C**), indicating MPTP/p induced the generation of p25. Next, we pretreated mice with PBS, AAV9-GFP, and AAV9-GFP-CIP via ICV injection 1 week before MPTP/p insult. One week after the last injection of probenecid, neuroprotection was assessed among four groups. As shown in **Figures 3D–F**, the number of TH positive neurons in the PBS group (86.17 ± 8.31, *n* = 6) was significantly less than control group (134.7 ± 12.06, *n* = 6, *p* < 0.01). CIP treatment protected TH positive neurons in CIP group (115.5 ± 6.93, *n* = 6, *p* < 0.05, compared to PBS group), while GFP treatment had no effect on DA neuroprotection (96.33 ± 9.36, n = 6, p > 0.05 compared to PBS group).

Hyperphosphorylation of MEF2D plays an important role in the pathologic progression of PD (Smith et al., 2006; Ke et al., 2015). MEF2D, a specific substrate of Cdk5/p25 (Ke et al., 2015), was highly phosphorylated in PBS and GFP groups. Importantly, the phosphorylation of MEF2D in CIP group was reduced when compared with PBS and GFP groups (**Figures 3G–J**). These data provided indirect evidence that hyperactivity of Cdk5/p25 was inhibited by CIP, which alleviated the loss of DA neurons induced by MPTP exposure.

### AAV9-GFP-CIP Alleviates Motor Symptoms and Anxiety-Like Behavior of the MPTP/p Induced PD Model Mice

**FIGURE 4:**
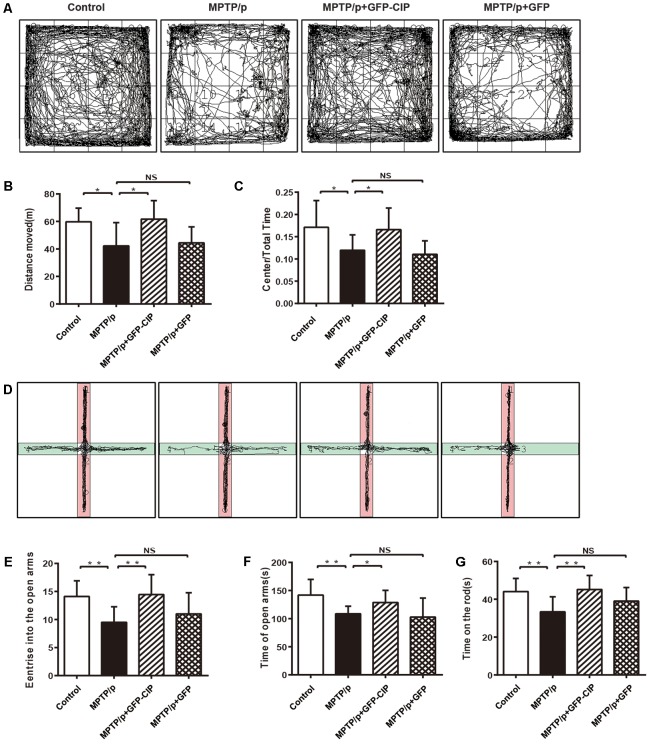
AAV9-GFP-CIP alleviated motor and non-motor symptoms in MPTP/p induced mouse model. Forty-eight mice were randomized into four treatment groups with 12 mice in each group. At the checking point, one mouse died in PBS or GFP group. **(A)** The track of different group mice in open field test was recorded for 30 min. **(B)** The total traveled distance of mice in each group in open field test. Total traveled distance of PBS group (42.25 ± 5.65) was shorter than the control group (59.75 ± 3.16, ^∗^*p* < 0.05). Total traveled distance of CIP group (61.68 ± 4.25) was longer than PBS group (^∗^*p* < 0.05) and there was no different between PBS group and GFP group, labeled NS. **(C)** The ratio of central distance of mice in each group in open field test. The ratio of central distance of PBS group decreased significantly, when compared with control group (^∗^*p* < 0.05). The ratio of central distance of CIP group was higher than PBS group (^∗^*p* < 0.05) and there was no different between PBS group and GFP group, labeled NS. **(D)** The track of four groups of mice in elevated plus maze was recorded for 5 min. **(E)** The times of each mouse entering the open arms in the elevated plus maze test. Mice of control group (14.10 ± 0.89) and CIP group (14.44 ± 1.18) entered the open arms more frequently, when compared with PBS (9.500 ± 0.98) and GFP (11.00 ± 1.19) groups (^∗∗^*p* < 0.01). **(F)** The duration of the mouse traveled in open arms. Mice of control group (141.9 ± 8.83) and CIP group (128.6 ± 7.20) spent more time in open arms when compared with PBS (108.6 ± 4.76) and GFP (102.7 ± 10.70) groups (^∗^*p* < 0.05). **(G)** The time of each mouse maintained on the rod was recorded. The graph shows the latency of mice falling off the rod. Mice in the CIP group (115.5 ± 6.93) maintained longer than in the PBS (86.17 ± 8.3) and GFP (96.33 ± 9.36) groups (^∗∗^*p* < 0.01). There was no difference between the control group (134.7 ± 12.06) and CIP group.

Next, we clarify whether DA neuroprotection by CIP after MPTP/p insult manifested by animal behavioral improvement. In this study, 12 mice were used for each group and the alive animals at the checking point were included in the final statistical analysis. We first observed the anxiety-like behavior among each group. Open field test and elevated plus maze were performed. The track records of four groups of mice in open field test and elevated plus maze are shown in **Figures 4A–F**.

In the open field test (**Figures 4A–C**), MPTP/p induced PD mice in PBS group traveled less distance and had lower ratio of central distance than the control group (*p* < 0.05), indicating that the general locomotor ability was damaged. Mice in CIP treated group traveled longer and had higher ratio of central distance than mice in PBS or GFP treated group (*p* < 0.05). No difference between the GFP and PBS groups was observed.

In the elevated plus maze (**Figures 4D–F**), PD model mice in PBS group preferred to stay longer in the closed arms and entered less in the open arms when compared with the control mice (*p* < 0.01), indicating that the PD mouse model had anxiety-like behavior. AAV9-GFP-CIP treated mice entered the open arms more frequently and spent more time in the open arms when compared with the PBS or AAV9-GFP treated mice (*p* < 0.05), while there had no difference between the GFP and PBS groups (*p* > 0.05).

Motor disturbance is another main compliant of PD patients and a good representative of the PD model mouse. As shown in **Figure 4G**, the rotarod test data indicated that mice exposed to MPTP/p induction fall down more quickly from the increasing rotating rod. AAV9-GFP-CIP treatment significantly improved motor symptoms in MPTP/p induced mice, although not completely recovered to the level in the control group; in contrast, AAV9-GFP treatment had no effect.

According to these data, we conclude that AAV9-GFP-CIP via ICV injection alleviates the motor and non-motor symptoms of the MPTP/p induced PD mice.

## Discussion

Evidence implies that the aberrant activity of Cdk5/p25 involves in PD (Camins et al., 2006). Peptides derived from p35, such as p5 or p10, selectively inhibit the hyperactivity of Cdk5/p25 *in vitro* or *in vivo* and prevent DA neuronal loss (Chew et al., 2010; Binukumar et al., 2015; Amin et al., 2016; Zhang et al., 2016a). Here we evaluate the neuroprotection of CIP in MPTP/p induced PD model mice. To assure long-term expression of CIP, we used vector AAV9 to deliver CIP via ICV in the mice brain 1 week before MPTP/p insult. Our data showed that AAV9-GFP-CIP succeeded in preventing DA neuronal loss in striatum, which accompanied by alleviation of the motor symptoms and anxiety-like behavior of mice.

In this study, we chose MPTP/p insulted mouse model other than other PD mouse models including BAC-α-Syn-GFP transgenic mice, Pitx3-mutant model mice, and MPTP -acute or sub-acute mouse model, since probenecid slows down the clearance of MPTP and entends duration of DA neurons damage; hereby it imitates most of behavioral symptoms and pathological changes in PD (Binukumar et al., 2015; Zhang et al., 2016b; Brandt et al., 2017; Zhao et al., 2017; Zhong et al., 2017). In MPTP/p induced PD mice (Jackson-Lewis and Przedborski, 2007; Meredith et al., 2008), DA neurons and spontaneous activity were significantly decreased. This chronic PD mouse model also displays anxiety-like behavior as indicated by the elevated plus maze assay. Different from the MPTP/p regimen described in Meredith et al. (2008), we injected MPTP by 10 consecutive days to replace the 3.5-day-interval injection in the present study. Similarly, DA neuronal loss, motor deficits, and neuropsychiatric phenotype were observed.

Hyperactivity of Cdk5/p25 contributes to the pathologic progression of several degenerative diseases by phosphorylation different relevant substrates, e.g., tau, neurofilament proteins, and β-amyloid precursor protein related to AD, MEF2D, synuclein, Parkin, and Prx2 related to PD (Su and Tsai, 2011). In MPTP/p induced PD mice, we observed remarkable elevation of the level of p25 at the check point, 1 week after the last probenecid treatment (**Figures 3A,C**). Although we were unable to check Cdk5/p25 activity due to the experimental material limitation, instead, we observed the phosphorylation staus of MEF2D, the specific substrate of Cdk5 (Smith et al., 2006; Ke et al., 2015). Increased level of phosphorylated MEF2D was found in the brain of model mice (**Figure 4G**), which indirectly suggested the increased activity of Cdk5. Since Cdk5/p25 hyperactivity involves in the progression of PD and the neurotoxicity of MPTP induced mice (Nakamura et al., 1997; Alvira et al., 2008; Sundaram et al., 2012), we evaluated whether the effects of CIP in MPTP/p induced PD model were based on the inhibition of Cdk5/p25 hyperactivity. In a previous study, we have demonstrated that AAV9 mediated CIP inhibits the activity of Cdk5/p25 complex indicating by reduction of phosphorylated tau and Aβ aggregation in AD mouse model (He et al., 2017). Similarly, in MPTP/p induced PD model, we demonstrated that pretreatment of AAV9-GFP-CIP significantly prevents DA neuronal loss and improves motor and non-motor symptoms of the disease mice. Moreover, AAV9-GFP-CIP decreased the level of phosphorylated MEF2D in PD mice, indicating inhibition of Cdk5/p25 hyperactivity. AAV9-GFP-CIP alleviated not only motor symptoms but also non-motor symptoms in MPTP/p induced PD model mice, suggesting that inhibition of hyperactivity of Cdk5/p25 by CIP might be a new strategy for the treatment of neurodegenerative diseases including AD and PD.

Compared to other p35-derived peptide, our study demonstrates that it is practical to deliver CIP by AAV9. Pretreatment by multiple injection of TFP5 inhibits Cdk5/p25 hyperactivity and protects against neurotoxicity induced by acute or sub-acute MPTP exposure (Binukumar et al., 2015; Zhang et al., 2016a). Compared to repeated injection, AAV9 mediated CIP required only one dose injection, since AAV9-GFP-CIP via ICV injection might provide neuronal degeneration as long as 8 months as checked in APP/PS1 mice (He et al., 2017). The next step is to evaluate the effect of AAV9-GFP-CIP after the onset of the symptoms and long-term effect in PD models. It will be also interesting to know whether AAV9-GFP-CIP provides neuroprotection in other PD mouse models.

In brief, our study provided further evidence of the effect of Cdk5 hyperactivity on pathogenesis of DA neuronal loss in MPTP/p induced PD model mice. AAV9-GFP-CIP via ICV injection expressed CIP widely in the brain of mice including midbrain, inhibited hyperphosphorylation of substrate of Cdk5/p25, alleviated the pathologic changes, and improved motor and non-motor symptoms in MPTP/p induced PD mouse model.

## Author Contributions

RH performed most experiments. WH contributed to histologic experiments and western blot analysis. YaoH did data analysis and drafted the manuscript. MX, PS, and YinH helped the animal behavioral study. HX co-designed the study. YafH co-designed the study, monitored the process of the experiment, and revised the manuscript.

## Conflict of Interest Statement

The authors declare that the research was conducted in the absence of any commercial or financial relationships that could be construed as a potential conflict of interest.
